# Foraging for high caloric anthropogenic prey is energetically costly

**DOI:** 10.1186/s40462-019-0159-3

**Published:** 2019-05-24

**Authors:** Susanne van Donk, Judy Shamoun-Baranes, Jaap van der Meer, Kees C. J. Camphuysen

**Affiliations:** 1 Department Coastal Systems, NIOZ Royal Institute for Sea Research and Utrecht University, P.O. Box 59, 1790 AB Den Burg, Texel, The Netherlands; 2 Theoretical and Computational Ecology, IBED, University of Amsterdam, Science Park 904, 1098XH Amsterdam, The Netherlands; 3 Department of Animal Ecology, VU University, De Boelelaan 1105, 1081 HV Amsterdam, The Netherlands

**Keywords:** Anthropogenic impact, Energy expenditure, Foraging strategies, *Larus argentatus*, Movement

## Abstract

**Background:**

Several generalist species benefit from food provided by human activities. Food from anthropogenic sources is often high in caloric value and can positively influence reproductive success or survival. However, this type of resource may require specific foraging skills and habitat experience with related costs and benefits. As a result, not all individuals utilize these resources equally, with some individuals preferentially foraging in habitats where natural resources of lower energy content are predominant, possibly due to lower energy expenditure of the specific foraging strategy.

**Methods:**

Here we investigate whether foraging in habitats which contain high caloric resources of anthropogenic origin is energetically costlier than foraging in habitats with low caloric resources such as intertidal areas or agricultural and natural areas, for example due to increased flight costs, in a generalist seabird, the herring gull *Larus argentatus*. We use data from GPS trackers with tri-axial acceleration measurements that allow us to quantify time-energy budgets, representing energy expenditure during foraging trips of herring gulls for each habitat.

**Results:**

We show that the rate of energy expenditure is on average 34% higher when individuals forage for high caloric prey in marine and urban areas compared to foraging for low caloric prey in intertidal and agricultural areas. Energetic estimates suggest that if birds would feed completely on these resources, they have to gather ~ 400 kJ per day more to compensate for the higher foraging costs.

**Conclusions:**

Energy expenditure differs among foraging habitat and may thereby influence foraging decisions of individual herring gulls. As management of anthropogenic resources changes, so too may the costs and potential benefits of foraging strategies which are strongly tied to human activities.

**Electronic supplementary material:**

The online version of this article (10.1186/s40462-019-0159-3) contains supplementary material, which is available to authorized users.

## Background

Many species experience a loss in resource availability due to human influences in their environment, but some species take advantage of resources that comes available due to human activities [1]. For example, predators such as red foxes *Vulpes vulpes* and coyotes *Canis latrans* have expanded their foraging activities to urban areas in recent decades to profit from anthropogenic resources [2–5]. Generalist species are especially suited to exploit human refuse, as they have a broad prey spectrum and exhibit flexibility in their behavior [5–10].

The resources or foraging patches individual animals choose to forage on may depend on the trade-off between costs and benefits of different foraging strategies [11–13]. For instance, some prey might have benefits like a high energetic value or they are beneficial for breeding success, but they might be energetically costly to forage on due to special foraging skills that need to be learned [14], a long searching or handling time [15] or a high level of predation [16] or competition [17].

A specific example of a generalist species that has to make foraging decisions in a landscape which has changed by humans is the herring gull *Larus argentatus*. Herring gulls have adapted their foraging behavior to human activities, and forage at refuse dump sites, waste treatment centers and on fisheries discards from commercial fisheries [18–21], profiting from relatively high caloric prey. Fishery discards and refuse were found to have a beneficial effect on reproductive success in several populations of gulls [19, 21–23], which suggests that animals foraging on these prey have a higher net energy intake and are thereby able to offer more food in terms of kilojoules to their offspring. The net energy intake is determined by the energy intake per unit time as well as the energetic costs of foraging per unit time [24], which might differ per foraging habitat. However, assessing and comparing foraging costs in terms of energy expenditure associated with different foraging habitats in the wild remains challenging. Even in relatively well studied species, such as the herring gull, little is known about the energetic investments in different foraging strategies and how differential costs may influence foraging decisions.

In this paper, we investigate whether energetic costs of foraging varies between different foraging habitats. We conducted our study in a population of herring gulls that is studied thoroughly over the last 10 years on the island of Texel, the Netherlands [25]. Intertidal areas provide bivalves, the predominant prey type for birds within this colony, but during chick rearing the diet becomes more diverse [23]. As shown in other gull studies [1, 26, 27], high caloric prey are important to ensure sufficient chick growth in this population [23]; breeding pairs that provision their chicks more regularly with refuse and fishery discards fledged more and larger chicks. Still, many individuals continue to forage mainly in intertidal areas even during chick rearing, suggesting that there are higher costs involved in foraging for the more beneficial high caloric prey (Table 1). We hypothesize that foraging for high caloric prey of anthropogenic origin is energetically more costly than foraging for the more predominant low caloric prey, as foraging for anthropogenic prey might require costly flight and competitive behavior. The higher energetic costs of foraging for anthropogenic prey may at least partially explain why some individuals prefer other foraging habitats.

**Table 1:** Overview of the diet of Herring Gulls breeding on Texel during the chick care of the breeding season

Habitat	Anthropogenic	Marine	Intertidal	Terrestrial	Other-Fresh water	Other-Colony
Frequency of occurrence	17.6%	33.2%	67.6%	9.9%	1.6%	8.0%
Number of samples	699	1319	2683	394	64	316
Common species/types	Plastic packaging (58%) Chicken (20%) Bread (16%) Pork (11%)	Flatfish (44%) Whitefish (37%) *Liocarcinus holsatus* (25%) *Crangon crangon* (20%) Small pelagics (12%)	*Mytilus edulis* (82%) *Carcinus maenas* (17%) *Ensis americanus* (11%) *Asterias rubens* (5%)	Insects (48%) Cattlefeed, grains (18%) Berries and seeds (17%) Earthworm (13%) Birds (13%) Rabbits & rodents (11%)	*Rutilus rutilus* (86%) *Perca fluviatilis* (9%)	*Larus* gull chicks (67%) *Larus* gull egg (32%)
Availability	Opening hours & waste cleaning operations	Nearby fishing fleets	Low tide	Variable	Unclear	Breeding season
Energetic value	Up to very high ~ 10–25 kJ/g	Moderate to high ~ 4–10 kJ/g	Low to moderate ~ 2–5 kJ/g	Variable ~ 2–9 kJ/g	Moderate ~ 4–6 kJ/g	Moderate ~ 4–8 kJ/g
Digestive constraint	Large bones, platics, metals, glass	Fish bones, scales	Breaking shells with muscular gizzard	Bones, fur, chitin	Fish bones, scales	Bones, down, eggshells

Frequency of occurrence of prey types are shown per foraging habitat based on all samples gathered (*n* = 3969), together with the most common species or prey types within each habitat groups. Furthermore, an indication is given about the availability, energetic value in kJ g^− 1^ wet weight and possible digestive constraints associated with these prey. The analysis was based on regurgitates gathered in the colony during fieldwork between 2006 and 2016, see for more information references [23, 25]

Bird-borne GPS trackers with tri-axial accelerometers, make it possible to measure behavior and estimate energy expenditure. Using more than 10 years of dietary data and color ring recordings, we link habitat use to prey types most likely to be acquired in each habitat (Table 1) [28, 29]. We tested whether the energy invested in foraging is higher in habitats containing prey of anthropogenic origin (marine and built up areas) than when foraging in habitats containing low caloric prey (intertidal areas and non-built up terrestrial areas) by comparing habitat use with energy expenditure during foraging trips. We quantified time energy budgets of herring gulls using GPS tracking and concomitant acceleration measurements. We show that more energy is invested in foraging in habitats with anthropogenic prey than when foraging in habitats with intertidal or terrestrial prey in our study system and we discuss the consequences of energetic costs of different foraging strategies in the context of a food landscape strongly influenced by humans considering what gulls have experienced over the past 40 years and what is expected in the coming decades.

## Methods

**Fig. 1:**
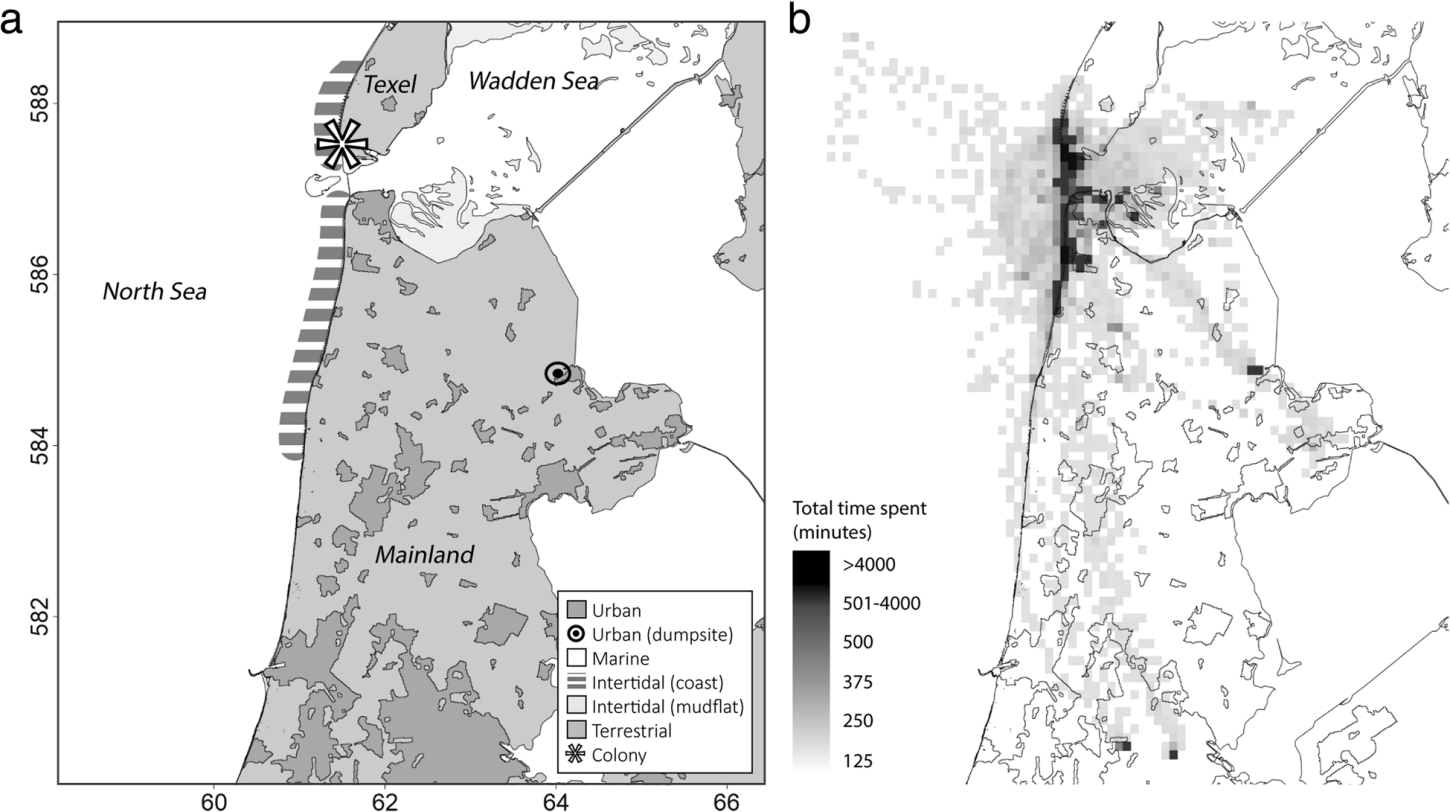
Map of the study system and habitat use (**a**) Overview of the study area, latitude and longitude are indicated on the x and y axis in UTM zone 31 (× 10.000), breeding colony is indicated with an asterix (615,201.4 E, 5873649 N). Foraging areas of category urban which contain refuse are indicated in dark grey. Another source of human waste is the closest waste treatment center to the colony, indicated with a circle. Foraging area of category marine with main prey fishery discards are North Sea and Wadden Sea. Foraging areas of category intertidal are indicated in the dashed area, but herring gulls also forage in the Wadden Sea on intertidal mudflats indicated in light grey. Terrestrial foraging areas are found on Texel and the mainland and are indicated in grey. **b** Space use of all the herring gulls used in the study (*n* = 17) during chick care in breeding seasons of 2013–2016, expressed in total time spent in minutes per square kilometer

The study was carried out between May 2013 and August 2016 at a breeding colony of approximately 4000 pairs of herring gulls which breed sympatrically with approximately 11,000 pairs of lesser black-backed gulls *Larus fuscus* at the island of Texel, the Netherlands (53°00′N, 04°43′E; 615,201.4 E, 5873649 N UTM zone 31; Fig. 1).

### GPS tracking

Thirty-one adult herring gulls (17 males and 14 females) were caught with walk-in traps during incubation between 2013 and 2015. Solar-powered GPS trackers of the UvA Bird Tracking System [30, 31] were mounted to the birds with a 3-g non-flexible Teflon harness on the back of the birds. As recommended for seabirds [32] GPS-tracker and harness together weighted less than 3% of the body mass of the birds which was on average 2.4% of female body mass and 2.1% of male body mass. These trackers measure, among others, the geographic location and time (UTC), and acceleration in three directions (surge, sway and heave). Tracking devices were calibrated to convert surge, sway and heave acceleration data to g-force (1 g_n_ = 9.81 m/s^2^). At time of capture, body mass (g), wing (mm), tarsus (mm), head (mm) and bill (mm) lengths were taken. The birds were sexed on the basis of head plus bill length (mm) [33]. Birds were released after attaching the GPS tracker and taking body measurements, which took approximately 20 min. The tracking frequency was set to every 10 min inside the breeding territory and every 5 min outside the breeding territory. As we had the possibility to change measurement frequency while the tracker was on the bird, we took occasionally higher resolution measurements. For better comparison, we resampled the data in this case to the standard measurement frequency. Tri-axial acceleration was periodically measured, only outside the breeding colony, at 20 Hz for 1 s directly following a GPS fix.

### Data selection and processing

For our analysis, we used GPS data of individuals that had a nest with chicks for at least 5 days after hatching of the first egg and we only used data up to 10 days after hatching to control for differences in demands when chicks grow bigger. We studied the costs of foraging during chick care, as prey choice have shown to be important for reproductive success in this period [23].

We compared the costs of foraging for different resources by analyzing foraging trips. We defined a foraging trip as a continuous period beginning when an individual travelled more than 100 m from its nest and ending when the individual returned to within 100 m of the nest. For each GPS location we attributed a ‘centered duration’ which was calculated by averaging the backward and forward time intervals between locations. The centered duration was used in further analysis to calculate trip duration and time spent in flight.

To calculate energy expenditure of foraging, we made use of acceleration data. Each acceleration measurement was attributed to one of 11 behaviors. We classified behaviors by training a random forest machine-learning algorithm for the classification of accelerometer data [31, 34]. We used two datasets to train the model on 11 different behaviors. The first is based on annotated accelerometer data of lesser black-backed gulls [34], which is a species that is comparable in size and morphology, and most behavior with the herring gull. The second dataset contained accelerometer data of herring gull specific foraging behavior which was annotated with synchronized video recordings [31]. The final random forest model used had an accuracy for predicting the 11 behaviors of 94%. The 11 behaviors were then aggregated into four behaviors, which are inactive behavior (sitting, standing or floating), terrestrial movement (terrestrial locomotion, looking and standing while looking for food, handling prey and other), soaring flight (soaring and maneuvering) and flapping flight (regular and extreme flapping flight).

Gaps in the GPS measurements occurred, and we excluded trips from analysis when gaps were bigger than 20 min when outside the breeding colony. Besides, we only used trips of which at least 80% of the GPS fixes were accompanied with acceleration measurements. After data selection*,* 605 trips were included in the analysis of 17 different individual herring gulls. One herring gull was included in the analysis for three consecutive years.

### Energetic costs

To compare energy expenditure of foraging in different habitat, we used three proxies for energy expenditure: (1) trip duration (h), (2) duration spent on flapping flight per trip (h) and (3) average estimated hourly energy expenditure per trip (kJ h^− 1^). Trip duration was calculated by summing all the ‘centered durations’ per trip.

The duration spent on flapping flight per trip was calculated by summing the ‘centered duration’ of measurements which were assigned to flapping flight. The duration of flapping flight per trip was used as a proxy for energy expenditure as flapping flight is thought to be the most energetically expensive form of locomotion compared to other behaviors [35].

We estimated the rate of energy expenditure per trip by estimating metabolic rates in kilojoules for the four classified behaviors per individual herring gull [31]. We calculated the basal metabolic rate (BMR) per individual in kJ day^− 1^ as 2.3 × body mass(g)^0.774^ at catching per individual (mean ± standard deviation of 19.64 ± 1.31 kJ h^− 1^ for all animals in our study) [36]. As the BMR does not account for thermoregulation when temperature is lower or higher than the thermo-neutral zone, digestion or little body movements, we calculated resting metabolic rate (RMR) as 1.7 × BMR [37, 38], with an average of 33.39 ± 2.22 kJ h^− 1^ over all individuals. For the energetic cost of the behavior ‘inactive’ we used RMR. We estimated the energetic cost of ‘terrestrial movement’ as 2 × BMR. This estimation was based on a formula of costs for terrestrial movement of Bautista et al. (1998) [39]; costs terrestrial movement (kJ day^− 1^) = (5.6 × W_kg_^0.246^ + 11.4 × W_kg_^-0.285^ × v) × 86.4, where W_kg_ is body mass (kg) and v is velocity in m s^− 1^. As v we used 0.4 m s^− 1^ which is the average velocity while walking of herring gulls with GPS trackers in this study. The formula of Bautista et al. is based on data of starlings *Sturnus vulgaris*, but two studies in barnacle geese *Branta leucopsis* show similar energy expenditure of terrestrial locomotion compared to basal metabolic rate [40, 41]. The cost of soaring flight was estimated as 2 × RMR [37] and the cost of flapping flight was estimated as 7 × RMR [35]. We calculated energy expenditure by summing the ‘centered duration’ for the four classified behaviors per trip and multiplying these with the energetic estimations (hour^− 1^) of these four behaviors. To calculate energetic costs per hour, we divided energy expenditure of the whole trip by its trip duration.

### Habitat use

To compare trips with different habitat use, we calculated the percentage of time gulls spent per trip in four foraging habitats which are termed (1) urban (2) marine, (3) intertidal and (4) terrestrial. We expect urban and marine environments to include predominantly high caloric prey (e.g. refuse and fishery discards) whereas intertidal and terrestrial include mainly low caloric prey such as bivalves and crabs and terrestrial invertebrates (Table 1)(Fig. 1).

To calculate habitat use, we took all the GPS positions outside the colony into account, apart from when animals are commuting (i.e. when an individual is flying in a straight line from one place to the other). To select the commuting GPS positions, we made use of an expectation maximization binary clustering for behavioral annotation developed by Garriga et al. 2016 [42]. This clustering algorithm uses turning angle and velocity obtained from successive locations to cluster GPS positions in four behavioral categories which are High velocity/Low turn (HL), High velocity/High turn (HH), Low velocity/Low turn (LL), Low velocity/High turn (LH). We assumed that an animal is commuting when velocity is high and turning angle low (HL category). We applied the clustering algorithm per individual in a given year using the r package *EmbC* and applied a pre-smoothing procedure which is provided by the packages to account for temporal associations.

Subsequently, we coupled every non-commuting GPS position to one of the four foraging habitats using several shapefiles of foraging areas around the colony and the behavioral classifications. We used the following shapefiles of foraging areas: North Sea and Wadden Sea, urban areas, breakwaters, beach & intertidal mudflats, agriculture & natural land (Additional file 1: Table S1). Often, the area around the breakwater, beach and intertidal mudflats is also available for foraging during low tide (personal observations) and therefore we also assigned GPS points which were assigned to North Sea or Wadden Sea closer than 50 m to breakwater, beach or intertidal mudflats to the intertidal habitat type. GPS positions where assigned to urban habitat when in urban areas. GPS positions where assigned to the marine when in the North Sea and Wadden Sea and on intertidal mudflats with the behavioral mode flying or floating. Almost all fish that the herring gulls of this colony consume originates from fishery discards [25] (p.337). GPS positions were assigned to intertidal habitat when on breakwaters and beach and on intertidal mudflats when the behavioral mode was resting or terrestrial movement which indicates foraging on intertidal mudflats. GPS positions where assigned to terrestrial habitat when in agricultural or natural areas.

### Statistical analysis

**Fig. 2:**
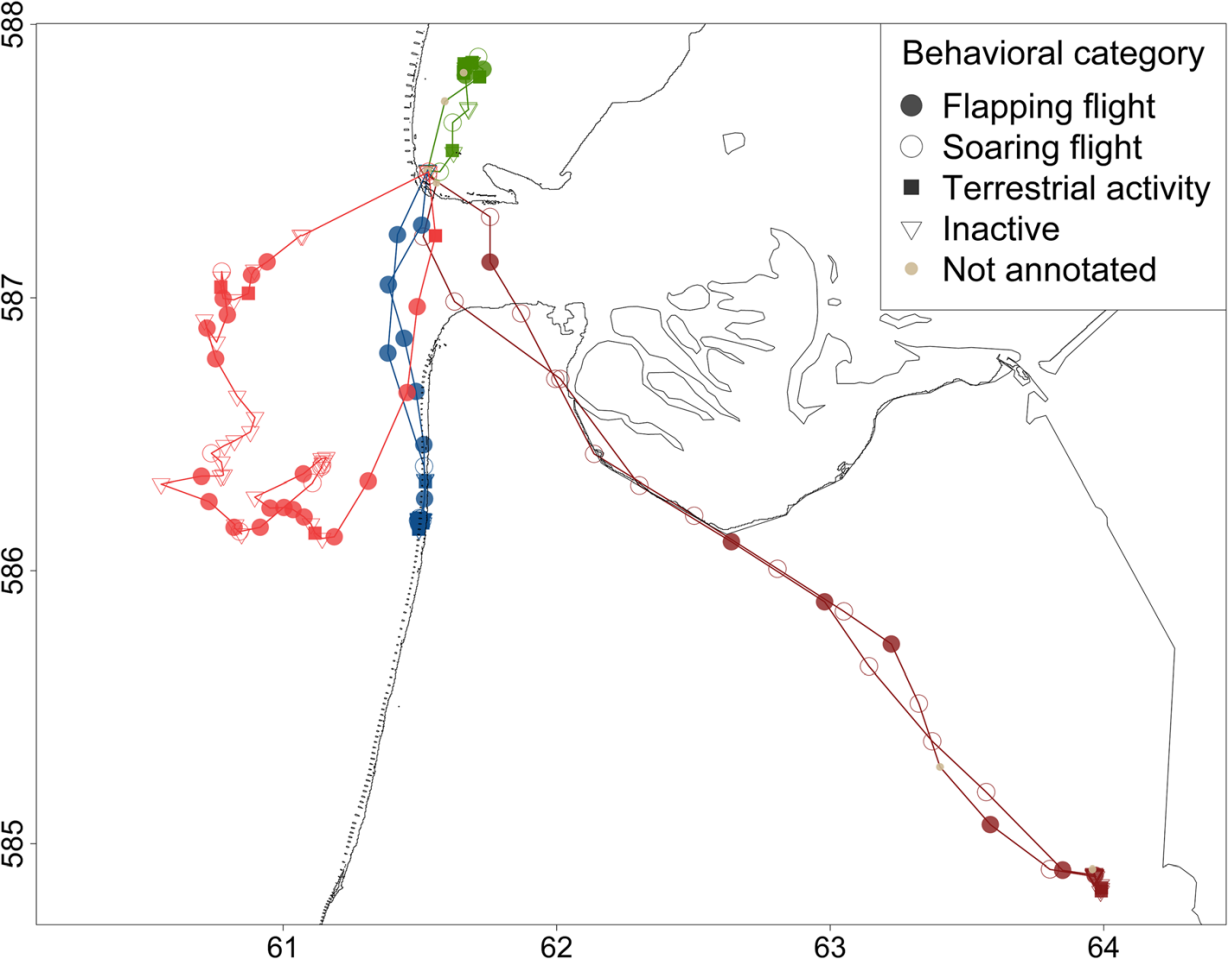
Examples of four foraging trips which are indicated in four different colors. Latitude and longitude are indicated on the x and y axis in UTM (× 10.000). Every point corresponds to one GPS fix and the shape of the points indicates the behavior of the animal (Flapping flight, soaring flight, terrestrial activity, inactive or not annotated). In dark red; a foraging trip of category urban. In light red; a foraging trip of category marine. In blue; a foraging trip of category intertidal. In green a foraging trip of category terrestrial

To test whether foraging costs differ depending on the foraging habitat, we divided all foraging trips in five habitat categories based on the time an animal spent in every foraging habitat. A foraging trip with 50% or more of its non-commuting GPS positions in one of the four foraging habitats, was assigned to category urban, marine, intertidal or terrestrial. A foraging trip with less than 50% of its non-commuting GPS positions in one of the four foraging habitats was assigned to a fifth category; mixed. Figure 2 shows examples of foraging trips of four categories.

We tested the hypothesis whether trips of the five habitat categories differed in energy expenditure by fitting linear mixed-effect models. We fitted a separate model for each of the response variables: trip duration in hours, duration spent on flapping flight in hours and rate of energy expenditure per hour. We included habitat categories as fixed effect and bird ID as random intercept in the models. Response variables in the models were transformed to obtain normality and homogeneity of variance; trip duration and energy expenditure were transformed with the natural logarithm, duration in flapping flight was transformed with the square root. Commuting GPS fixes were included in the calculation of these response variables. The models were tested against a null model which only contained the random factor. When the model was significantly better than the null model, we performed post hoc Tukey testing for the fixed part of the model to test which habitat category differed using the lsmeans function from the lmer Test library [43]. *P*-values, ∆AICc and the model estimates and standard errors were reported, as well as marginal and conditional R squared values for mixed models [44]. Although we did not have specific hypotheses about the role of sex, mass or sampling year in this study, we did explore these factors in the models by comparing the residuals of the model with sex, sampling year and mass. After this analysis, we concluded that we could ignore these factors in our analyses.

## Results

### Habitat use and foraging trips

During the non-commuting phase of foraging trips, herring gulls spent most time in areas with relatively lower caloric prey; most time was spent in the intertidal habitat followed by terrestrial habitat (Table 2). Less time was spent in areas with relatively high caloric prey; gulls spent from these habitat types a bit more time in marine habitat than in urban habitat (Table 2). As a result, the number of foraging trips per category were also not evenly distributed; most trips were assigned to intertidal and least to category mixed (Table 2).

**Table 2:** Habitat use outside the breeding colony of non-commuting GPS data of all herring gulls used in this study (*n* = 17) and foraging trip details (*n* = 605)

Habitat	Time (h)	Time (%)	Nr. trips	Mean trip duration	Proportion flapping trip ^−1^	Rate of energy expenditure	Total energy
Urban	155	14	66	4.02 ± 2.28	0.25 ± 0.11	89.41 ± 21.88	357.17 ± 205.50
Marine	199	18	86	2.66 ± 1.46	0.34 ± 0.17	100.70 ± 33.97	261.52 ± 152.22
Intertidal	535	48	271	2.78 ± 1.70	0.17 ± 0.11	69.88 ± 21.78	196.65 ± 127.88
Terrestrial	232	21	127	2.01 ± 1.67	0.17 ± 0.17	71.96 ± 34.63	146.89 ± 138.62
Mixed	–	–	55	4.00 ± 2.23	0.24 ± 0.12	85.10 ± 23.27	323.52 ± 180.79

Total time in hours (Time (h)) and percentages (Time (%)) of non-commuting GPS data of all herring gulls used in this study, the number of trips per habitat category (Nr. Trips), mean ± standard error of the trip duration per habitat category (Mean trip duration; h), the proportion of flapping flight per trip per habitat category (Proportion flapping trip^−1^) and the average energy expenditure per time (Rate of energy expenditure; kJ h^− 1^) and per trip (Total energy; kJ) per habitat category

To test whether habitat use differed in energy expenditure per trip, we made use of three proxies for energy expenditure which were trip duration, time spent on flapping flight and energy expenditure for which we also included the time spent on commuting. Duration of foraging trips varied widely (range: 0.6–14.4 h, mean ± SE: 2.8 ± 1.9 h) and mean foraging duration was highest for urban and mixed foraging trips. Similarly, we found high variation in the proportion of time spent on flapping flight per trip (range: 0–1 per h trip, mean ± SE: 0.21 ± 0.15 h) and the estimated energy spent per trip (range: 11 - 1006 kJ, mean ± SE: 224 ± 163 kJ) and they were both highest for urban trips. But the estimated energy spent per hour (range: 26 - 252 kJ, mean ± SE: 78 ± 29 kJ) was highest for marine foraging trips. Mean trip duration, time spent on flapping and the estimation of energy expenditure per trip were lowest for terrestrial trips, but estimated energy expenditure per hour was lowest for both terrestrial and intertidal trips.

### Link between energetic costs and habitat use during foraging trips

**Fig. 3:**
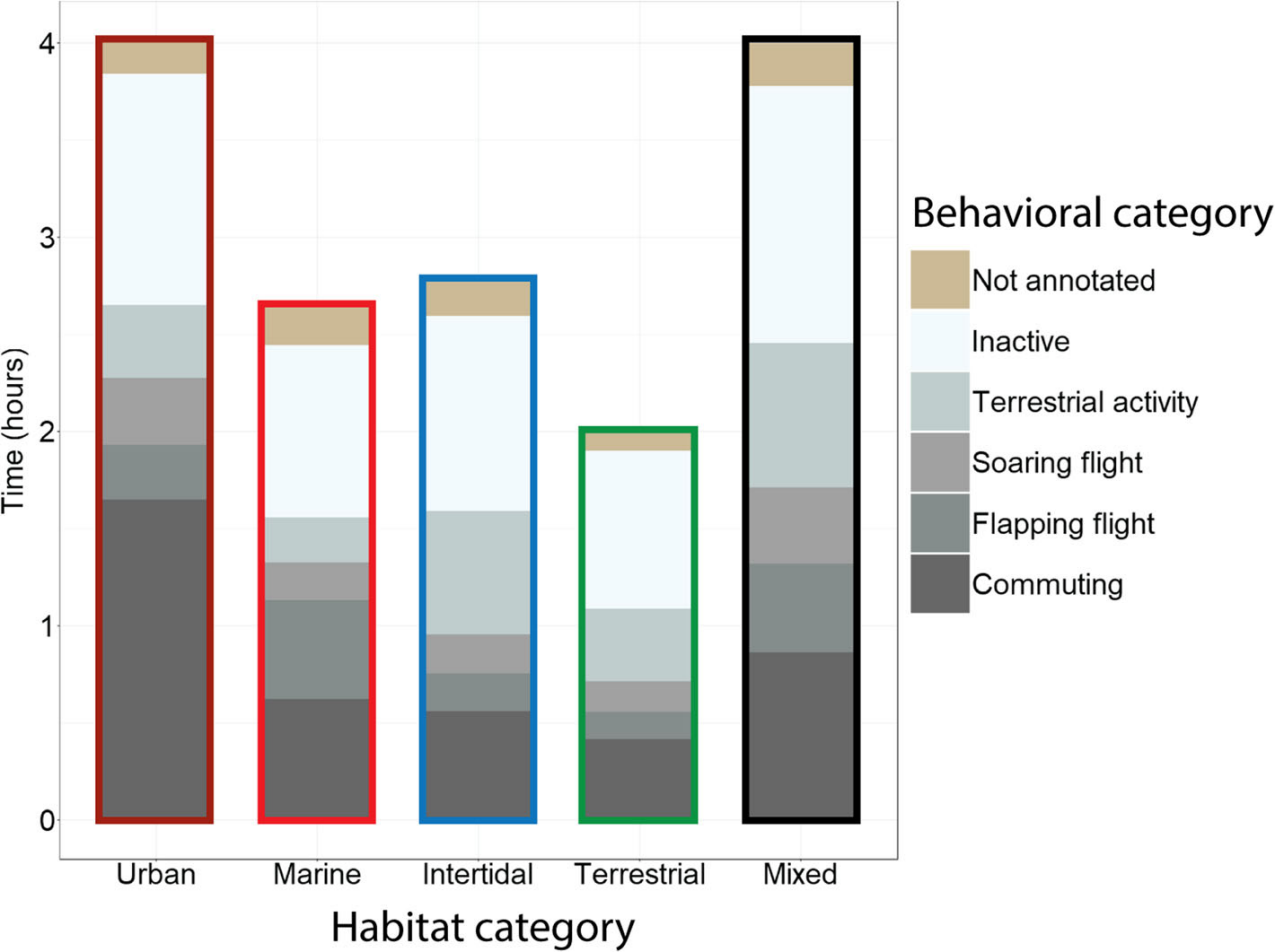
Trip duration and time spent (h) on the different behavioral categories per habitat category (*n* = 605 foraging trips). Trips on which individuals spent more than 50% in one habitat were assigned to main habitat category urban, marine, intertidal or terrestrial. Trips on which individuals spent less than 50% in one habitat were assigned to main habitat category mixed

**Fig. 4:**
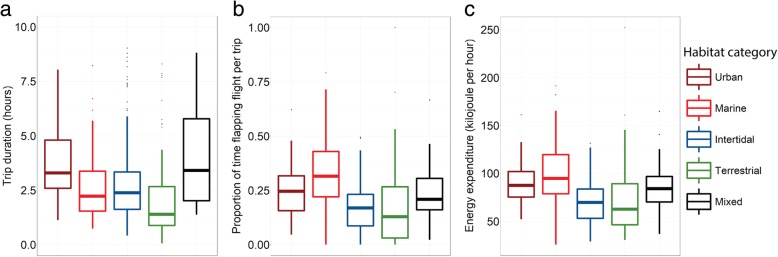
The relationship between habitat use per habitat category and energetic costs. The boxplots show the median values of energetic costs per habitat category. **a**-**c** represents habitat category in relation to (**a**) trip duration in hours (**b**) Proportion of time in flapping flight and (**c**) energy expenditure in kilojoules per hour

Foraging costs were also significantly higher in urban and marine habitats than foraging in intertidal or terrestrial foraging habitats, while mixed foraging trips had similar energetic costs as trips to urban and marine habitat (Table 3). Duration of trips differed between the categories; urban and mixed trips were significantly longer than trips to marine and intertidal habitat and trips of category terrestrial were shorter than trips of all other categories (Fig. 4a). But the higher energetic costs of urban, marine and mixed trips are mainly caused by relatively more time spent on flapping flight per trip (Figs. 3 and 4). More specifically, the trips in category urban, marine and mixed included more time spent on flapping flight and a higher estimated energy expenditure per hour than trips in category intertidal and terrestrial (Table 3; *p* < 0.05), while energy expenditure per hour did not differ between intertidal and terrestrial trips (Fig. 4b and c).

**Table 3:** Model results of the relationship between habitat use and response variables the logarithm of trip duration in hours (Log (Duration)), the square root of duration of flapping flight in hours (sqrt (Flapping)), and the logarithm of energy expenditure in kJ per hour (log (energy))

Response variable	Fixed factors	Model estimates	Chisq	∆AICc	p-value	df	R^2^m	R^2^c
Log (Duration)	**Intercept U.**	**1.22 ± 0.09** ^**a**^	89.55	81.55	<2e-16	4	0.13	0.23
	Marine	−0.40 ± 0.10^b^						
	Intertidal	−0.35 ± 0.09^b^						
	Terrestrial	− 0.77 ± 0.09^c^						
	Mixed	−0.06 ± 0.11^a^						
Sqrt (Flapping)	**Intercept U.**	**0.92 ± 0.05** ^**a**^	120.58	112.58	<2e-16	4	0.18	0.25
	Marine	−0.06 ± 0.05^a^						
	Intertidal	−0.30 ± 0.05^b^						
	Terrestrial	−0.43 ± 0.05^c^						
	Mixed	−0.05 ± 0.06^a^						
Log (Energy)	Intercept U.	4.46 ± 0.04^a^	96.20	88.20	<2e-16	4	0.15	0.16
	**Marine**	**0.09 ± 0.06** ^**a**^						
	Intertidal	−0.27 ± 0.05^b^						
	Terrestrial	−0.29 ± 0.05^b^						
	Mixed	−0.05 ± 0.06^a^						

We used linear mixed-effect models with habitat category as fixed effect and bird ID as random intercept. Model estimates and standard error (SE) are shown for the five categories; Intercept U is the intercept and the estimate for category urban. When the estimates for the other fixed factors are negative, this category has a lower output of the response variable than the Intercept U. When the estimates of the other fixed factors are positive, this category has a higher output of the response variable than the Intercept U. The fixed factor with the highest model estimate per model is printed in bold and the statistical differences of the groups are indicated with letters. We provided the marginal (R^2^m) and conditional (R^2^c) for every model which represents, respectively, the variance explained for the fixed factors alone or the variance explained taking by both the fixed and random factors (individual birdIDs)

## Discussion

By comparing foraging trips of different habitat use, we found, in line with our expectations, that foraging at sea for discards or in dumps or cities where most human refuse is obtained was energetically costlier (about 34 higher costs per hour) than foraging in other habitats. Mixed foraging strategies (the use of more foraging habitats) had a similar energy expenditure as foraging trips towards anthropogenic resources (urban and marine trips). Higher costs resulted in particular from the time spent in flapping flight, but mixed and urban foraging trips were also longer in duration. Foraging in terrestrial or intertidal habitats was comparable in energetic costs per hour, but foraging in terrestrial habitat was least costly per trip, because the duration of these trips was shorter. We discuss the consequences of these foraging costs considering the past and future changes in the food landscape and costs and benefits of prey.

We used rough estimates for energy expenditure in this study, and compare them with other studies to determine whether our estimates were biologically meaningful. These studies described below measured food intake or energy expenditure of adult seabirds. A study on lesser black-backed gulls in captivity measured a fish intake of 900–1400 kJ per day [45] and with an assimilation efficiency of 75% [46], these gulls used 28–44 kJ per hour. This gull species is smaller than the herring gull and the birds were not able to show energetically costly behavior like searching for food and flying. Another study on black-legged kittiwakes *Rissa tridactyla*, seabirds which are half the weight of herring gulls, found that birds used 41 kJ per hour during foraging trips [47]. Our estimations of an average energy use of 78 kJ per hour during foraging trips seem to be quite reasonable, compared to measurements from other seabirds.

Foraging for anthropogenic resources is energetically expensive in our system but how big are these costs in terms of quantity of prey? The average length of discarded flatfish that is found in the breeding colony is 12–13 cm with an energetic value of 84 kJ per fish [48, 49]. Daily energetic costs per day for an animal solely foraging for fishery discards are 1462 kJ per day, assuming 14 h in the colony spending 33 kJ per hour and 10 h outside the colony spending 100 kJ per hour (Table 2). The average assimilation efficiency is 75% [46], so such an animal has to catch 23 fishes (1949 kJ) for its own subsistence. The amount of prey that it catches should be higher, as birds also have to gather food for chicks. Compared to an animal foraging solely in intertidal or terrestrial habitat which costs about 70 kJ per hour (daily costs about 1162 kJ), it would need to catch 5 flatfish more per day to compensate for the higher foraging costs.

When animals forage for anthropogenic prey, they have to catch more prey to compensate for higher foraging costs. However, the differences in energetic costs per hour between resting metabolic rate and foraging are in fact considerably larger, respectively 33 kJ and 70–100 kJ depending on foraging habitat (Table 2). The total time a gull spends on foraging might actually be more important in terms of its energetic costs than in which habitat a gull forages. A gull that mostly forages in urban or marine habitat could compensate for its higher foraging costs by conducting fewer foraging trips per day and can thus have similar daily costs compared to a gull that mostly forages in terrestrial or intertidal habitats. Among individuals in our study, this does not seem to be the case. The average number of trips per day differs considerably per individual, but individuals that forage more for anthropogenic resources do not have less trips per day (Additional file 1: Table S1). Interestingly, there does seem to be a correlation between the number of trips per day and the time spent in intertidal area. Individuals that spent most of their trips in intertidal areas seem to have a higher amount of trips per day compared to individuals that do not forage often in intertidal habitat (Additional file 1: Table S2 and Figure S1), suggesting that the low hourly costs of foraging in intertidal area are cancelled out by foraging more times a day. This relationship is mainly caused by two individuals, so a larger sample size would be needed to investigate this relationship more thoroughly.

Individual animals have to make foraging decisions based on the advantages and disadvantages between different foraging strategies [11, 50, 51]. One of the main benefits of foraging for refuse and fishery discards for herring gulls is the high caloric value per gram prey which helps reaching energetic demands of growing chicks (Table 1) [19, 21–23]. We found that herring gulls spent more energy to obtain these high quality prey by making longer trips and flying more (Table 2). That central-place foragers like breeding gulls spent more energy to obtain prey of higher quality has been found before. For example, Ring-billed gulls *Larus delawarensis* travelled further for foraging patches that provided higher mean energy intake, like refuse dumps [11], while foraging patches with lower mean energy intake, like agricultural fields, were only visited closer to the breeding colony.

To understand the net energy gain, it is also important to learn about the food intake per unit time. Although we have indirect proof that animals foraging for high caloric prey like fishery discards and human waste have a higher net energy gain, because of their better growing and surviving chicks [23], we miss a direct measurement of food intake per unit time. In future, we hope to be able to measure food intake per habitat by using detailed accelerometer data and video recordings of the different habitats. Another possibility to estimate energy intake is to use the dynamic energy budget model (DEB) [52]. This model can be used to estimate energy intake of growing chicks based on their weight, which will give a more precise estimate of prey intake in terms of kilojoules brought to the nest, even on a daily basis [53].

Characteristics of prey other than energetic gain and loss are also important for gulls, like the availability and predictability of prey (Table 1). Prey in terrestrial habitat are not always available during the breeding season. Earthworms, for example, are only available when the soil is moist, and agricultural areas provide most food when farmers are ploughing [11, 54, 55]. This might explain the shorter time herring gulls spent in this habitat, despite the low foraging costs, compared to intertidal foraging habitat (Table 2; 21% of foraging time). On the contrary, prey of the intertidal habitat (mostly bivalves) are very predictable (during low tide) and available in large amounts every day which might be the reason that most of the foraging time is spent in this habitat.

**Fig. 5:**
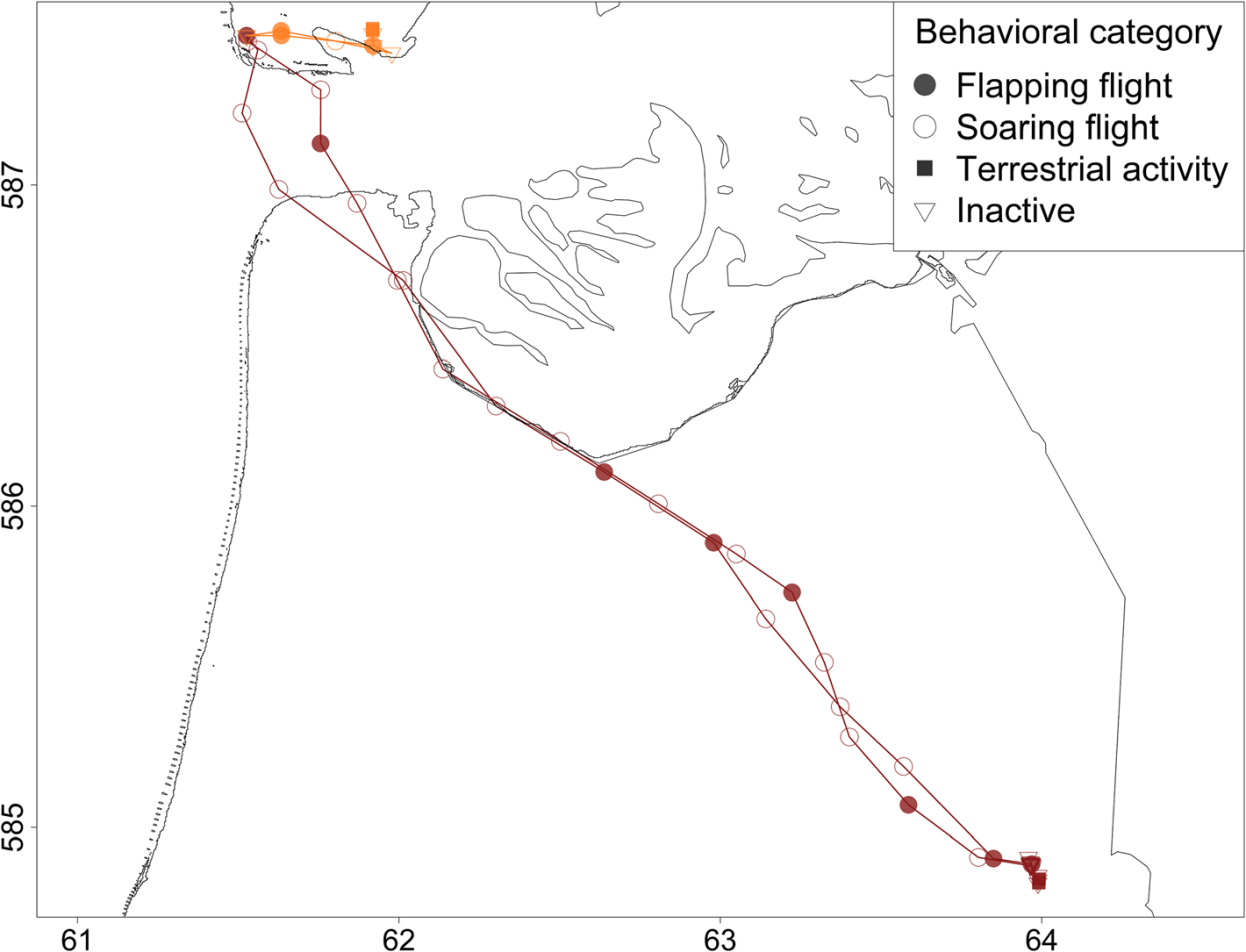
Comparison between imaginary foraging trip to the closest refuse dump 30 years ago (4 km distance of the colony; in orange) and a foraging trip to the closest waste treatment center (35 km distance of the colony; in dark red). Latitude and longitude are indicated on the x and y axis in UTM (× 10.000). Breeding colony is situated at 615201.4 E, 5873649 N UTM. Every point corresponds to one GPS fix and the size of the points indicates the behavior of the animal (resting, terrestrial locomotion, soaring flight or flapping flight). Estimated energetic costs of these trips of an animal with a body mass of 1000 g are respectively 160 kJ and 362 kJ

Although herring gulls have been benefiting from human resources, this situation is changing and resources have been decreasing the last decades. National and international legislation caused the decrease of the number of refuse dumps and the amount of refuse in most European countries including the Netherlands [25, 56–58]. Furthermore, fishing fleet densities in the region have decreased and legislation towards discarding bycatch became stricter [59–62]*.* Resource availability for the herring gulls breeding on Texel have changed in a similar way as the number of fishing vessels and refuse dumps in the close surroundings decreased a lot [22, 25]. Only 30 years ago, there was still a refuse dump within 4 km of the breeding colony (Fig. 5), whereas now the closest waste treatment center is 35 km away. Unfortunately, we cannot compare foraging costs between the past and now, but using the knowledge we have gained in the present study, we could compare an hypothetical foraging trip to the disappeared refuse dump with a foraging trip to the closest waste treatment center now, which is presented in Fig. 5. When we assume that foraging time is similar between the two places (about 1.5 h), the costs of foraging on refuse dumps might have more than doubled for these gulls due to the increased cost of flight. In future, the amount of refuse and fishery discards will probably be even less. Fishing effort is still decreasing and a more sustainable way of fishing is in development [63]*.* This will probably increase time and energy needed for foraging because of higher competition.

If the foraging costs for these resources become higher than the reproductive gains, gulls might have to change their behavior. Gulls could decrease foraging costs, for instance by decreasing the distance that they have to commute between breeding territory and foraging areas by breeding closer to urban areas or refuse dumps. Gulls do breed on rooftops in increasing numbers, with sometimes higher breeding performance [64–67]. Although the foraging behavior of these gulls is largely unknown, the proximity of foraging possibilities to a colony does affect the composition of the diet of that colony [68–70]. A recent study on breeding colonies of herring gulls in the USA, showed that birds breeding closer to urbanization had shorter foraging trips [70]. But whether gulls will move between breeding locations is questionable, as gulls of this colony rarely move to another breeding colony, even though breeding success is low [25] (p.326). The location of the breeding site on Texel does not seem very favorable anymore in terms of food sources compared to 30 years ago [22]. Still, there are no indications of a decrease in breeding pairs over the last decade. Whether individual herring gulls of this colony will adapt their behavior is unclear, but these human induced changes in the environment create a natural experiment to study the effect on animals’ behavior. In future, we could use these environmental changes to look into whether individuals will respond to these changes by adapting their foraging or breeding behavior.

## Conclusions

We studied costs of foraging in different habitats in the herring gull, to get a better understanding of the factors that can shape animals’ foraging decisions in an environment which is highly affected by humans. We found that foraging for high caloric prey of anthropogenic origin is costly in terms of foraging effort compared to other foraging options, but these prey are beneficial for chick growth and survival. Foraging for less beneficial prey for reproduction, like terrestrial and intertidal prey, were less costly in terms of foraging effort. Recent and future alterations in fishery discards and garbage management will increase foraging costs of these prey, which will affect the balance between costs and benefits. Gulls might have to adapt or change foraging or breeding behavior, although it is not clear whether they will be able to do this due limited flexibility.

## Additional file

Additional file 1: **Table S1.** for shapefiles used to determine habitat use. **Table S2** and **Figure S1** for showing the relationship between the total time foraging and habitat use per individual. (PDF 195 kb)
