# Supplementary material for: Foraging for high caloric anthropogenic prey is energetically costly
Source: Mov Ecol. 2019 May 24;7:17. doi: 10.1186/s40462-019-0159-3 (PMC6533676; doi:10.1186/s40462-019-0159-3)
Supplement: Supplementary file 1 — Table S1. for shapefiles used to determine habitat use. Table S2 and Figure S1 for showing the relationship between the total time foraging and habitat use per individual. (PDF 195 kb) [file 40462_2019_159_MOESM1_ESM.pdf]

Additional file 1

Table S1 Habitat types and shapefiles used in the analysis.

**Habitat**	**Shapefile**
North Sea and Wadden Sea	GDAM January 2012 European boundaries polygon (Hijmans, 2012)
Urban areas	OpenStreetMap data (OpenStreetMap contributors, 2017)
	CORINE Urban morphological zones 2006 (European Environment Agency (EEA), 2017)
Beach	LGN5 (Landelijke Grondgebruikskartering 5) (Hazeu, 2005)
Breakwaters	Delineated based on QGIS openlayer plugin google earth (QGIS Development Team, 2009)
Tidal flats	Rijkswaterstaat (http://www.rijkswaterstaat.nl)
Agricultural and natural	OpenStreetMap data (OpenStreetMap contributors, 2017)
	Netherlands Natural Features shapefile (MapCruzin, 2017)

Table S2 Average number of foraging trips per individual bird ID per habitat category and the total number of trips per day. The table is ordered in ascending order of total number of trips per day. The highest value per individual (favored habitat category) is underlined.

	**Average number of trips per day**					
**Bird ID**	*Urban*	*Marine*	*Intertidal*	*Terrestrial*	*Mixed*	*Total*
6217	0.72	0.31	0.72	0.72	0.62	3.10
6206	0.96	0.48	1.44	0.24	0.72	3.83
1600	0.00	0.91	2.73	0.13	0.13	3.90
6004	0.56	0.42	1.25	1.11	0.56	3.90
6006	1.95	0.00	0.43	1.19	0.33	3.90
6009	0.00	0.42	1.58	2.00	0.00	4.00
6076	0.10	0.50	1.80	0.90	0.70	4.00
6073	0.00	0.99	1.44	1.66	0.11	4.20
6080	1.56	0.78	0.39	1.17	0.39	4.29
6014	0.80	1.60	0.40	1.00	0.60	4.40
6205	0.00	0.39	2.70	0.64	0.77	4.50
6210	1.07	0.59	2.13	0.36	0.36	4.50
6202	1.02	0.87	1.60	0.87	0.44	4.80
6015	1.61	1.38	0.69	1.15	0.00	4.83
6071	0.00	0.83	2.50	1.04	0.52	4.90
6016	0.05	0.56	3.63	0.87	0.31	5.42
6208	0.00	0.63	4.28	0.32	0.48	5.70

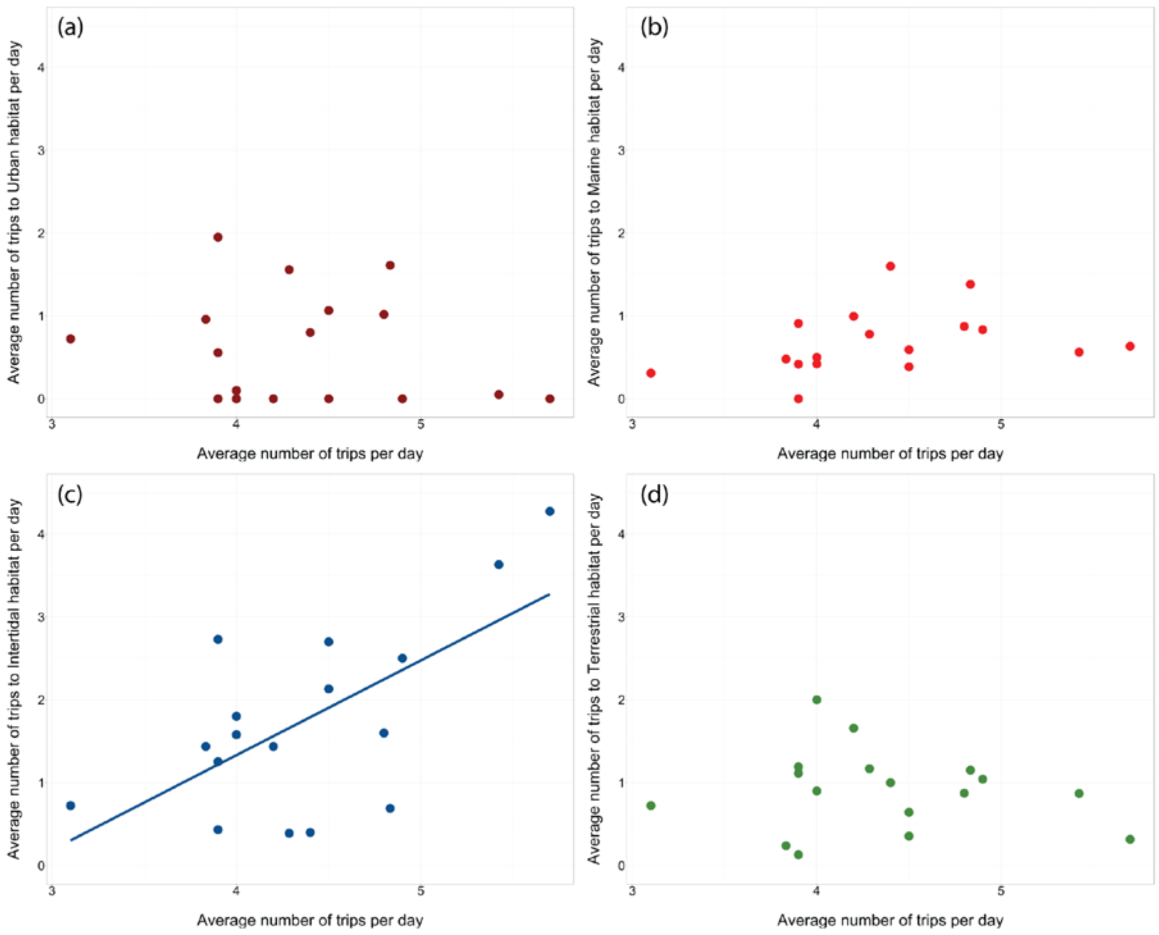

Figure S1 Relationship between average number of trips per day on the x-axis and average number of trips to the specific habitats per day per bird ID on the y-axis per bird-ID. Every point represents one Bird ID. Figures (a)-(d) show average trips per day against average trips to (a) urban habitat, (b) marine habitat, (c) intertidal habitat and (d) terrestrial habitat. Only the relationship between average number of trips per day and average number of trips to intertidal habitat is statistically significant (linear model intercept= 3.74±0.23, estimates = 0.36±0.11, p=0.006, R^2=0.37).
